# Supplementary material for: Overlapping upstream ORFs ending at c.125 lead to reduced Endoglin, contributing to Hereditary Hemorrhagic Telangiectasia
Source: Commun Biol. 2025 Jul 18;8:1072. doi: 10.1038/s42003-025-08461-6 (PMC12274349; doi:10.1038/s42003-025-08461-6)
Supplement: Supplementary file 2 — Supplementary Information [file 42003_2025_8461_MOESM2_ESM.pdf]

Overlapping upstream ORFs ending at c.125 lead to reduced Endoglin, contributing to Hereditary Hemorrhagic Telangiectasia

Carole Proust[1], Clémence Deiber[1], Caroline Meguerditchian[1], Maud Tusseau[2], Alexandre Guilhem[3,4,5], Shirine Mohamed[6], Aurélie Goyenvalle[7], Béatrice Jaspard-Vinassa[8], Sophie Dupuis-Girod[2,9], David-Alexandre Trégouët[1*], Omar Soukarieh[1,8 *]

[1] Univ. Bordeaux, INSERM, Bordeaux Population Health Research Center, UMR 1219, F-33000 Bordeaux, France
[2] Hospices Civils de Lyon, French National HHT Reference Center and Genetics department, Hôpital Femme-Mère-Enfant, 69677, Bron, France
[3] Hospices Civils de Lyon, Service de Génétique, Groupement Hospitalier Est, 69677, Bron, France
[4] Centre de Référence National pour la maladie de Rendu-Osler, Groupement Hospitalier Est, Bron, France
[5] TAI-IT Autoimmunité Unit RIGHT-UMR1098, Université de Bourgogne, INSERM, EFS-BFC, Besançon, France.
[6] Département de Médecine interne et Immunologie Clinique, CHRU BRABOIS, Vandoeuvre-lès-Nancy, France
[7] Université Paris-Saclay, UVSQ, Inserm, END-ICAP, Versailles, France
[8] Univ. Bordeaux, INSERM, Biology of Cardiovascular Diseases, U1034, F-33600 Pessac, France
[9] Univ. Grenoble Alpes, Inserm, CEA, Laboratory Biology of Cancer and Infection, F-38000 Grenoble, France

[*] Joint corresponding authors: david-alexandre.tregouet@u-bordeaux.fr; omar.soukarieh@inserm.fr
Phone: +33 5 57 89 01 06; Fax: na

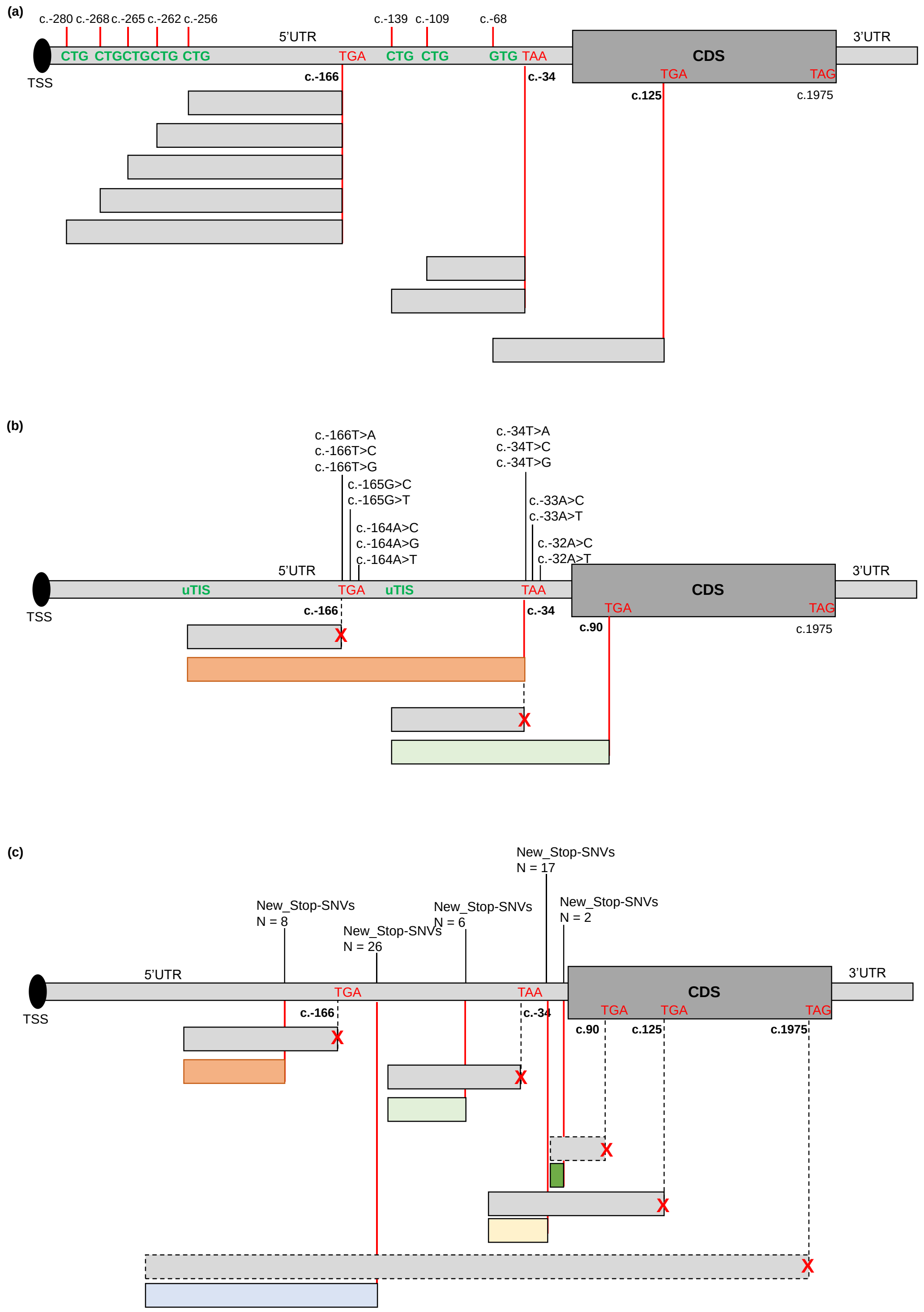

Supplementary Figure 1. Illustration of naturally existing upstream Open Reading Frames (upORFs) (a) uStop-deleting variants (b) and new-Stop-creating variants in the 5'UTR of ENG. The type of upstream Translation Initiation Site (uTIS) and position of stop codons associated with the existing or the new upORFs are indicated. The red cross represents the deleted uStop replaced by another one downstream (b) or the shortening of existing upORFs by the creation of upstream stop codons. The number of variants creating stop codons in the same frame is indicated. TSS, translation start site; CDS, CoDing Sequence; SNVs, single nucleotide variants.

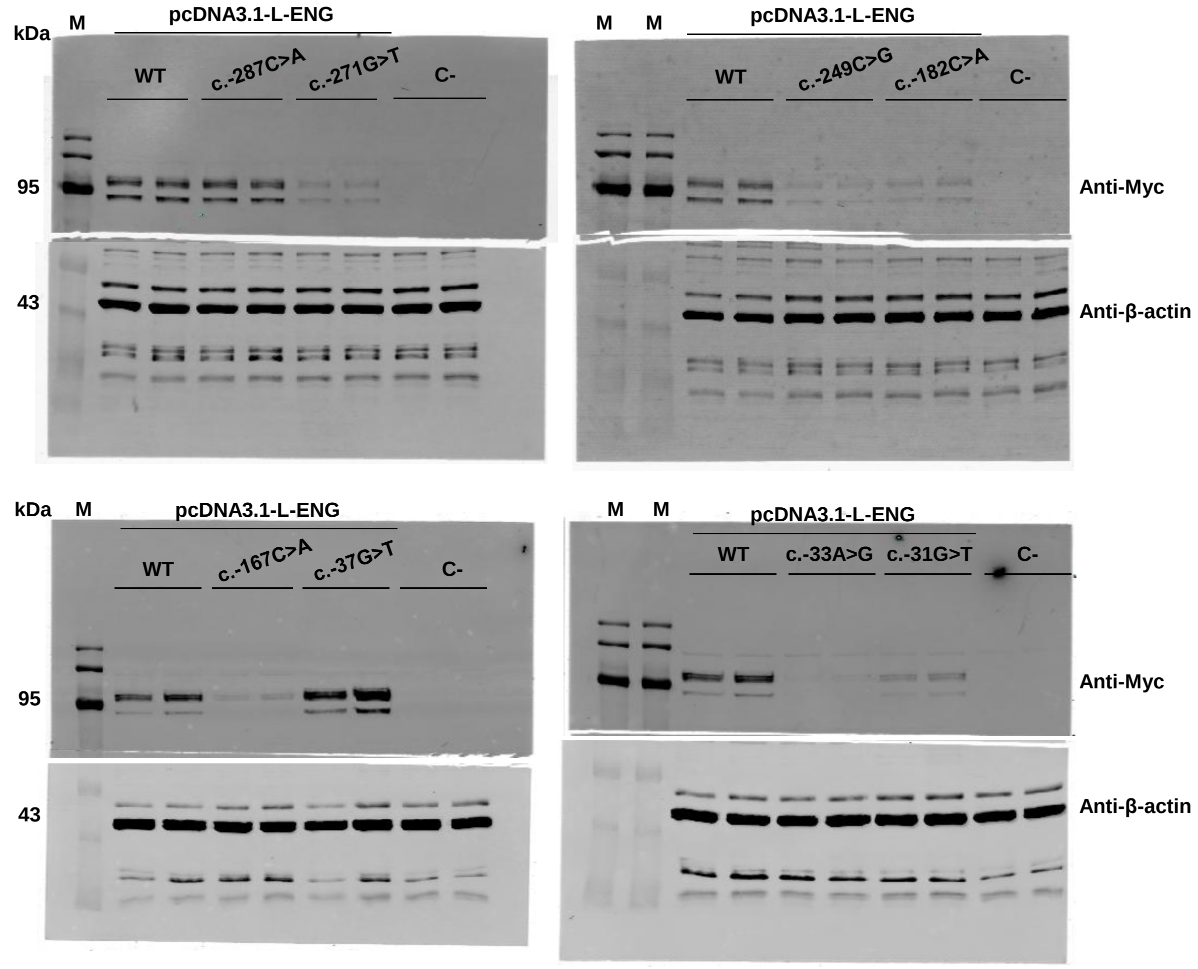

Supplementary Figure 2. Western blot results on total proteins extracted from transfected HeLa cells with 1 µg of pcDNA3.1-L-ENG constructs containing *ENG* variants at the origin of uAUGs in frame with the same stop codon at position c.125. The blots correspond to the uncropped upper and lower blots in main Figure 2b. Each blot was cut in 2 parts and each part was incubated with the corresponding antibody: monoclonal anti-(c-Myc Tag) or anti-β-actin as a loading control. Specific fluorescent secondary antibodies were used. Finally, Odyssey Infrared Imaging System (Li-Cor Biosciences) in 700 and 750 channels was used to scan, reveal, and quantify the blots. HeLa cell extracts were analyzed in duplicates. kDa, kilodalton; M, protein ladder; WT, wild-type, C-, negative control corresponding to pcDNA3.1- empty vector.

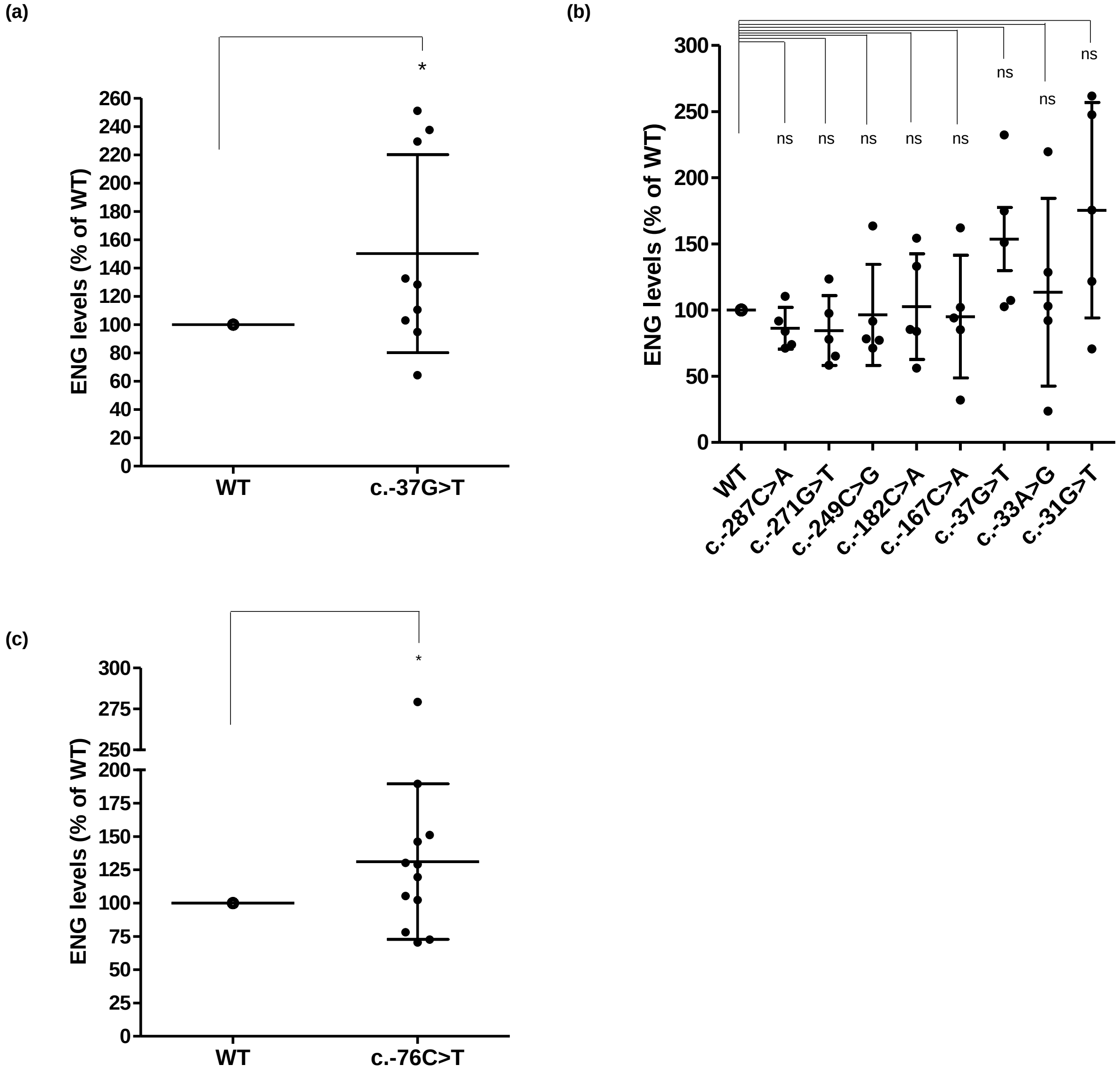

Supplementary Figure 3. Quantification of ENG levels in HeLa cells from Western blot (a) and RT-qPCR (b & c) analyses. (a) ENG steady-state level in HeLa cells is significantly increased with the c.-37G>T variant in comparison to the wild-type (WT) construct. For quantification, the average of the duplicate has been calculated from the quantified values and ENG levels have been normalized to the corresponding β-actin levels then to the WT (%). The two bands obtained for the Endoglin, corresponding to the more glycosylated (upper band) and less/non glycosylated (lower band) ENG monomers, were taken together for the quantification. The graph with standard error of the mean is representative of 9 independent experiments. (b and c) RNA levels of Endoglin in wild-type and variant contexts. Normalized 2-ΔΔCT to the WT are shown. Graphs with standard error of the mean are representative of 5 (b) and 12 (c) independent experiments. *, p-value < 0.05; ns, non-significant (Kruskal-Wallis followed by Dunn test for multiple comparisons of variants versus WT, when needed).

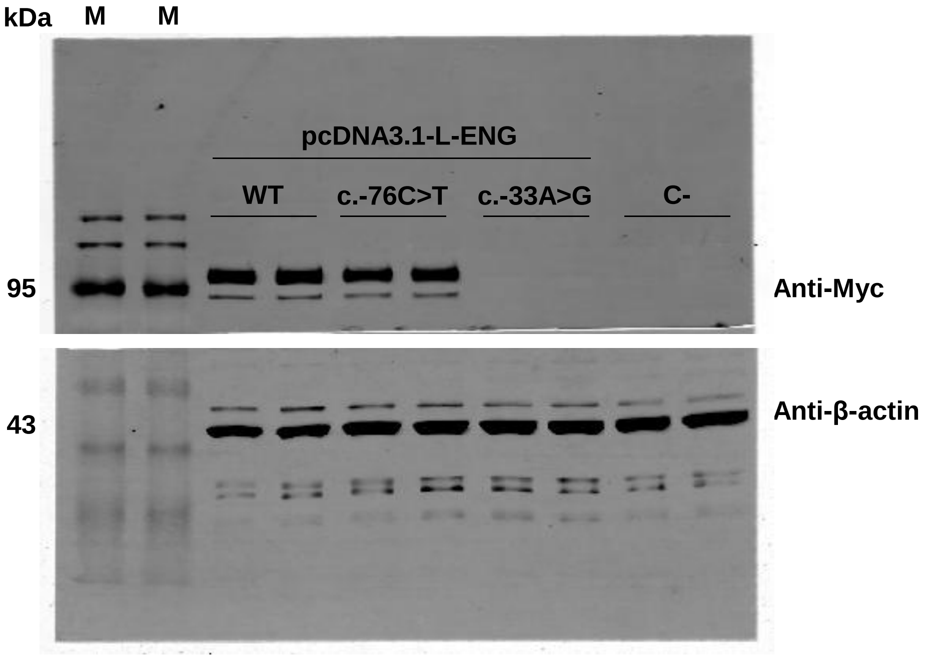

Supplementary Figure 4. Western blot results on total proteins extracted from transfected HeLa cells with 1 µg of pcDNA3.1-L-ENG. The blots correspond to the uncropped upper and lower blots in main Figure 3b. Each blot was cut in 2 parts and each part was incubated with the corresponding antibody: monoclonal anti-(c-Myc Tag) or anti-β-actin as a loading control. Specific fluorescent secondary antibodies were used. Finally, Odyssey Infrared Imaging System (Li-Cor Biosciences) in 700 and 750 channels was used to scan, reveal, and quantify the blots. HeLa cell extracts were analyzed in duplicates. kDa, kilodalton; M, protein ladder; WT, wild-type, C-, negative control corresponding to pcDNA3.1- empty vector.

References

1. Lind L, Ärnlöv J, Lindahl B, et al. Use of a proximity extension assay proteomics chip to discover new biomarkers for human atherosclerosis. *Atherosclerosis*. 2015;242(1):205–210.
2. Kozak M. Point mutations define a sequence flanking the AUG initiator codon that modulates translation by eukaryotic ribosomes. *Cell*. 1986;44(2):283–292.
3. Kozak M. An analysis of 5’-noncoding sequences from 699 vertebrate messenger RNAs. *Nucleic Acids Res*. 1987;15(20):8125–8148.
