# Supplementary material for: Overlapping upstream ORFs ending at c.125 lead to reduced Endoglin, contributing to Hereditary Hemorrhagic Telangiectasia
Source: Commun Biol. 2025 Jul 18;8:1072. doi: 10.1038/s42003-025-08461-6 (PMC12274349; doi:10.1038/s42003-025-08461-6)
Supplement: Supplementary file 3 — Description of Additional Supplementary Files [file 42003_2025_8461_MOESM3_ESM.docx]

**Description of Additional Supplementary Files**

Supplementary Data 1. Generated vcf file containing the totality of single nucleotide

variants (n = 909) in the 5'UTR of ENG (ENST00000373203.9; NM_001114753.3).

Supplementary Data 2. Single nucleotide variants in the 5'UTR of ENG (ENST00000373203.9; NM_001114753.3) annotated with MORFEE to: create new upstream Translation Initiation Sites (uTIS), create new upstream stop codons (New_Stop), and/or detele upstream stop codons (uStop). The 14 variants creating overlapping upstream Open Reading Frames ending with the stop codon at position c.125 and experimentally characterized are bolded.

Supplementary Data 3. Single nucleotide variants with multiple consequences on upstream Open Reading Frames in the 5'UTR of ENG (ENST00000373203.9; NM_001114753.3).

Supplementary Data 4. Single nucleotide variants creating upstream Translation Initiation Sites (uTIS) in the 5'UTR of ENG (ENST00000373203.9; NM_001114753.3) and reported in ClinVar <https://www.ncbi.nlm.nih.gov/clinvar/>. Variants were classified below according the type of the generated upstream Open Reading Frame.

Supplementary Data 5. Single nucleotide variants creating upstream Translation Initiation Sites (uTIS) in the 5'UTR of ENG (ENST00000373203.9; NM_001114753.3), extracted from MORFEEdb and reported in GnomAD V4 0.0 database (https://gnomad.broadinstitute.org/) . Variants were classified below according the type of the generated upstream Open Reading Frame.

Supplementary Data 6. Single nucleotide variants creating 2 upstream Translation Initiation Sites (uTIS) in the 5'UTR of ENG (ENST00000373203.9; NM_001114753.3).

Supplementary Data 7. Raw data associated with main Figures 2 and 3 and Supplemental Figure 3. Eight supplementary files are submitted in the same excel file (supplementary information). (Separate file)

Supplementary Data 8. Clinical data for ENG 5’UTR variants identified in HHT patients and experimentally analyzed in this study.

Supplementary Data 9. Details about primers used in this study. Restriction sites in cloning primers are underlined. Variations in directed mutagenesis (DM) primers are in lowercase.
