# Supplementary material for: Overlapping upstream ORFs ending at c.125 lead to reduced Endoglin, contributing to Hereditary Hemorrhagic Telangiectasia
Source: Commun Biol. 2025 Jul 18;8:1072. doi: 10.1038/s42003-025-08461-6 (PMC12274349; doi:10.1038/s42003-025-08461-6)
Supplement: Supplementary file 6 — Reporting Summary [file 42003_2025_8461_MOESM6_ESM.pdf]

nature portfolio

Corresponding author(s): DBPR COMMSBIO-25-3615-T

Last updated by author(s): Jun 16, 2025

Reporting Summary

Nature Portfolio wishes to improve the reproducibility of the work that we publish. This form provides structure for consistency and transparency in reporting. For further information on Nature Portfolio policies, see our Editorial Policies and the Editorial Policy Checklist.

Statistics

For all statistical analyses, confirm that the following items are present in the figure legend, table legend, main text, or Methods section.

n/a	Confirmed	
☐	☒	The exact sample size (*n*) for each experimental group/condition, given as a discrete number and unit of measurement
☐	☒	A statement on whether measurements were taken from distinct samples or whether the same sample was measured repeatedly
☐	☒	The statistical test(s) used AND whether they are one- or two-sided<br>*Only common tests should be described solely by name; describe more complex techniques in the Methods section.*
☐	☒	A description of all covariates tested
☐	☒	A description of any assumptions or corrections, such as tests of normality and adjustment for multiple comparisons
☐	☒	A full description of the statistical parameters including central tendency (e.g. means) or other basic estimates (e.g. regression coefficient) AND variation (e.g. standard deviation) or associated estimates of uncertainty (e.g. confidence intervals)
☐	☒	For null hypothesis testing, the test statistic (e.g. *F*, *t*, *r*) with confidence intervals, effect sizes, degrees of freedom and *P* value noted<br>*Give P values as exact values whenever suitable.*
☒	☐	For Bayesian analysis, information on the choice of priors and Markov chain Monte Carlo settings
☒	☐	For hierarchical and complex designs, identification of the appropriate level for tests and full reporting of outcomes
☒	☐	Estimates of effect sizes (e.g. Cohen's *d*, Pearson's *r*), indicating how they were calculated

Our web collection on statistics for biologists contains articles on many of the points above.

Software and code

Policy information about availability of computer code

Data collection	The used version of MORFEE tool is available at https://doi.org/10.5281/zenodo.14864790.
Data analysis	The used version of MORFEE tool is available at https://doi.org/10.5281/zenodo.14864790.

For manuscripts utilizing custom algorithms or software that are central to the research but not yet described in published literature, software must be made available to editors and reviewers. We strongly encourage code deposition in a community repository (e.g. GitHub). See the Nature Portfolio guidelines for submitting code & software for further information.

Data

Policy information about availability of data

All manuscripts must include a data availability statement. This statement should provide the following information, where applicable:

- Accession codes, unique identifiers, or web links for publicly available datasets
- A description of any restrictions on data availability
- For clinical datasets or third party data, please ensure that the statement adheres to our policy

ENG constructs generated during this study are available upon request by email from the corresponding author (omar.soukarieh@inserm.fr).
All raw experimental data analysed in this work are provided in Supplementary Table 7.

Research involving human participants, their data, or biological material

Policy information about studies with human participants or human data. See also policy information about sex, gender (identity/presentation), and sexual orientation and race, ethnicity and racism.

Reporting on sex and gender	The 2 ENG variants (c.-76C>T and c.-33A>G) identified in HHT patients we investigated in our study were found in female patients from the French National reference center for HHT.<br><br>Of note, the upORF-SNVs we investigated in this study as well as all the statistical and experimental investigations hold for both genders.
Reporting on race, ethnicity, or other socially relevant groupings	No categorization variables have been used.
Population characteristics	We identified 2 uTIS-creating variants, c.-33A>G and c.-76C>T, in unrelated HHT patients the French National reference center for HHT. The first one, creating a uAUG predicted to generate a uoORF ending at the c.125 codon and never reported in public databases, was identified in a patient with definite HHT according to Curaçao criteria.<br>The second variant, creating a non-canonical TIS (uCUG), is also predicted to generate a uoORF ending at the c.125 codon. This variant (rs943786398) was detected in 2 unrelated patients with unlikely HHT and has been classified as VUS in ClinVar. The proband in the first family had an atypical presentation for HHT with stroke and deep vein thrombosis associated with few telangiectasias. In the second family, the proband presented with pulmonary AVM and the father was an asymptomatic carrier of the variant.
Recruitment	Recruited as part of a molecular diagnosis routine conducted at the genetics department of the French National reference center for HHT (Lyon, France).
Ethics oversight	No patient material has been used in this study.

Note that full information on the approval of the study protocol must also be provided in the manuscript.

Field-specific reporting

Please select the one below that is the best fit for your research. If you are not sure, read the appropriate sections before making your selection.

☒ Life sciences ☐ Behavioural & social sciences ☐ Ecological, evolutionary & environmental sciences

For a reference copy of the document with all sections, see nature.com/documents/nr-reporting-summary-flat.pdf

Life sciences study design

All studies must disclose on these points even when the disclosure is negative.

Sample size	na
Data exclusions	na
Replication	All experiments have been performed in duplicate and all experiments have been repeated at least 3 times as indicated in the main text and in supplemental data.
Randomization	This is not relevant to our study. We performed cellular-based assays and compared samples in the same conditions each time.
Blinding	This is not relevant to our study. We performed cellular-based assays and compared samples in the same conditions each time.

Reporting for specific materials, systems and methods

We require information from authors about some types of materials, experimental systems and methods used in many studies. Here, indicate whether each material, system or method listed is relevant to your study. If you are not sure if a list item applies to your research, read the appropriate section before selecting a response.

Materials & experimental systems

n/a	Involved in the study
☐	☒ Antibodies
☐	☒ Eukaryotic cell lines
☒	☐ Palaeontology and archaeology
☒	☐ Animals and other organisms
☐	☒ Clinical data
☒	☐ Dual use research of concern
☒	☐ Plants

Methods

n/a	Involved in the study
☒	☐ ChIP-seq
☒	☐ Flow cytometry
☒	☐ MRI-based neuroimaging

Antibodies

Antibodies used	Mouse purified monoclonal anti-(c-Myc Tag) antibody (Merck Millipore, clone 9E10 | 05 419) and anti-β-actin (b-Actin (13E5) Rabbit mAb, Cell Signaling)
Validation	Anti-(c-Myc Tag) antibody is reactive in all species as reported at the manufacturer website.<br>Anti-β-actin: validated on different human extracts in Western blot by the manufacturer.

Eukaryotic cell lines

Policy information about cell lines and Sex and Gender in Research

Cell line source(s)	HeLa, epithelial human cells isolated from a cervical carcinoma derived from a 31-year-old patient (ATCC).<br>Human Umbilical Vein Endothelial Cells (HUVEC) from pooled donors (Lonza).
Authentication	None of the cell lines used were authenticated.
Mycoplasma contamination	All cell lines were tested negative for mycoplasma contamination.
Commonly misidentified lines (See ICLAC register)	None of the cell lines used were misidentified.

Clinical data

Policy information about clinical studies

All manuscripts should comply with the ICMJE guidelines for publication of clinical research and a completed CONSORT checklist must be included with all submissions.

Clinical trial registration	This is not a clinical trial.
Study protocol	Non applicable.
Data collection	Clinical data were communicated by the genetics department of the French National reference center for HHT (Lyon, France).
Outcomes	Clinical diagnosis of HHT is determined based on the Curaçao criteria established by the HHT international committee.

Plants

Seed stocks	Non applicable.
Novel plant genotypes	Non applicable.
Authentication	Non applicable.
